# Therapeutic plasma exchange in postpartum HELLP syndrome: a case report

**DOI:** 10.1186/s40981-023-00602-2

**Published:** 2023-02-20

**Authors:** Nana Kojima, Kosuke Kuroda, Makiko Tani, Tomoyuki Kanazawa, Kazuyoshi Shimizu, Jota Maki, Hisashi Masuyama, Hiroshi Morimatsu

**Affiliations:** 1 Department of Anesthesiology and Resuscitology, Okayama University Hospital, 2-5-1 Shikatacho, Kitaku, Okayama 700-8558 Japan; 2 Department of Anesthesiology, Kochi Health Sciences Center, Kochi, Japan; 3 Department of Obstetrics and Gynecology, Okayama University Hospital, Okayama, Japan

**Keywords:** HELLP syndrome, Postpartum, Plasma exchange therapy, Thrombotic microangiopathy

## Abstract

**Background:**

Postpartum hemolysis, elevated liver enzymes, and low platelet count (HELLP) syndrome is more difficult to treat than HELLP syndrome during pregnancy. We describe a case of postpartum HELLP syndrome that responded to plasma exchange (PE) therapy.

**Case presentation:**

A 30-year-old primipara woman was hospitalized for gestational hypertension at 33 weeks of gestation and underwent an emergent cesarean section at 36 weeks and 6 days of gestation due to rapidly progressing pulmonary edema. After delivery, liver dysfunction and a rapid decrease in platelet count were observed, and the patient was diagnosed with severe HELLP syndrome. She experienced multiple organ failure despite intensive care, and PE therapy was initiated. Her general condition dramatically stabilized within a few hours of PE therapy.

**Conclusion:**

It is controversial whether PE therapy should be used primarily in the management of HELLP syndrome, but early initiation of PE therapy could be effective for severe HELLP syndrome.

**Supplementary Information:**

The online version contains supplementary material available at 10.1186/s40981-023-00602-2.

## Background

Hemolysis, elevated liver enzymes, and low platelet count (HELLP) syndrome is one of the most serious complications in pregnancy. HELLP syndrome develops after delivery in 30% of the cases, with the majority developing within 48 h postpartum [1]. Postpartum HELLP syndrome is more severe and difficult to treat than HELLP syndrome during pregnancy [1]. Furthermore, there are only a few treatments for severe postpartum HELLP syndrome [2]. Herein, we describe a case of postpartum HELLP syndrome that responded to plasma exchange (PE) therapy.

## Case presentation

**Fig. 1:**
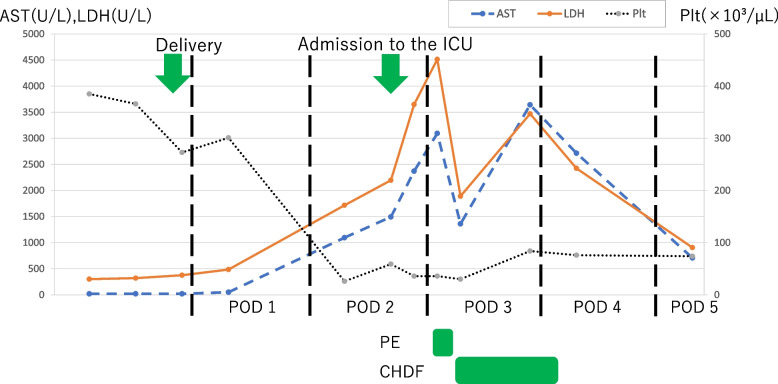
Changes in laboratory findings and events. After delivery, acute elevations in AST and LDH and a rapid decrease in platelet count were observed and continued to worsen. PE therapy was performed, and the laboratory findings improved. AST, aspartate aminotransferase (IU/L); LDH, lactate dehydrogenase (IU/L); Plt, platelet count (/L); PE, plasma exchange; CHDF, continuous hemodiafiltration; POD, postoperative day

The patient was a 30-year-old primipara with 150 cm height and 61.8 kg weight, and had good fetal growth. The patient was hospitalized for gestational hypertension at 33 weeks of gestation with blood pressure of 140/93 mmHg, which was refractory to magnesium sulfate. At 36 weeks and 6 days of gestation, due to rapidly progressing pulmonary edema and renal impairment with a serum creatinine of 0.90 mg/dL (Supplemental Figure S1), cesarean section was performed under spinal anesthesia with hyperbaric bupivacaine 10 mg and fentanyl 25 µg for pregnancy termination. After cesarean delivery, oliguria unresponsive to fluid loading persisted. On postoperative day 2, liver dysfunction with acute elevations in aspartate aminotransferase (AST) to 1096 U/L and lactate dehydrogenase (LDH) to 1717 U/L, and a rapid decrease in platelet count to 26,000/µL with decreased levels of fibrinogen and antithrombin III were observed (Fig. 1, Supplemental Table S1). The patient was diagnosed with a severe HELLP syndrome (class 1) (Supplemental Table S2) [3] and transferred to the intensive care unit (ICU) for strict management.

Her general condition deteriorated despite treatment with dexamethasone 6.6 mg every 12 h for three times, continuous nicardipine infusion, fluids, appropriate blood transfusion, and antithrombin III supplementation, and she developed to multiple organ failure and became drowsy. On computed tomography scan, hepatic infarction, intracranial hematoma, or cerebral hemorrhage were excluded, and a large volume of ascites and bilateral pleural effusions were observed. Eight hours after admission to the ICU, she was confused in the conversation and fell into delirium. Although her condition deteriorated, including state of consciousness, circulation, and hepatic and renal insufficiency, tracheal intubation or catecholamines were not required. However, due to her rapid deterioration and no improvement in her hematologic findings, PE therapy was initiated.

PE therapy was performed using 4800 mL of fresh frozen plasma (approximately 1.5 times the plasma volume), followed by continuous hemodiafiltration for oliguria. The patient's hemodynamic and mental status stabilized within a few hours of PE therapy. The day after PE therapy, laboratory findings and urine output improved, and hemodiafiltration was terminated. Both liver and kidney functions improved, and the patient was discharged to the general ward on day 7 of the ICU stay.

## Discussion

HELLP syndrome (hemolysis, thrombocytopenia, and elevation of bilirubin, LDH, and liver enzymes, low platelet count) is a life-threatening pregnancy complication. Early diagnosis is critical because serious illness and even death can occur in about 1.1% of cases [1]. HELLP syndrome is described as a complication of hypertensive disorders of pregnancy (HDP) [4], and 80% of patients with HELLP syndrome are complicated by HDP [5]. Symptoms of HELLP syndrome include eclampsia, disseminated intravascular coagulation (DIC), placental abruption, renal failure, and pulmonary edema [5, 6]. In this case, she had HDP, and had pulmonary edema. HELLP syndrome had to be taken into account. Martin et al. found that serious maternal complications, such as maternal death, convulsions, pulmonary edema, acute renal failure, acute hepatic failure, pulmonary hemorrhage, DIC, and stroke were found in 44% of patients in class 1 of the Mississippi classification (Supplemental Table S2) [3], a severity score, 13% in class 2, and 23% in class 3 [7]. In this case, all criteria regarding the platelet count, AST, and LDH were class 1, and the progression of the disease developed rapidly, that we considered the need for intensive care.

Delivery is the first-line management for HELLP syndrome. Most cases showed an improvement in laboratory parameters within 72 h after delivery [2]. However, if there is no improvement in laboratory data after 72 h of delivery, or if there is a worsening trend of the disease, including organ failure, the outcome would be considered poor [2], and more strict management is needed. Therapeutic options for severe HELLP syndrome are prophylaxis for eclampsia, DIC treatment, corticosteroid therapy, and PE therapy [8]. Although prophylaxis for eclampsia and DIC treatments are recommended in severe HELLP syndrome, whether PE therapy should be used primarily to manage HELLP syndrome remains controversial [8, 9]. PE therapy is suggested to be beneficial when HELLP syndrome persists for > 72 h after delivery [2, 10]. A previous report comparing patients with HELLP syndrome with and without PE therapy showed that the mortality was significantly lower, and ICU stay was significantly shorter in patients with PE therapy [10]. On the other hand, early PE therapy within 24 h postpartum has also been reported to be effective for patients with HELLP syndrome class 1 [11]. Furthermore, a practical plan for trial-PE has been suggested for cases of HELLP syndrome with multiple organ failure or extremely abnormal laboratory findings [12]. In our case, the patient had postpartum HELLP syndrome that developed more than 24 h after delivery, and treatment other than termination was required. After admission to the ICU, intensive care was continued, and corticosteroids were administered; however, there was no improvement in the patient's condition or laboratory data. Therefore, PE therapy was initiated. We decided to perform PE therapy within 24 h after the diagnosis of severe HELLP syndrome and 8 h after the ICU admission. After PE therapy, the patient's general condition stabilized immediately, and laboratory data improved. The patient had been treated with several drugs, all of which may have been effective. And, since the patient’s condition improved within 72 h of cesarean delivery, it is possible that the improvement was due to the delayed effect of the termination. However, early initiation of PE therapy might have been effective for early recovery.

The mechanism of PE therapy in HELLP syndrome remains unclear [8]. HELLP syndrome is considered a microangiopathic disease and is included in thrombotic microangiopathy (TMA); therefore, it may work by removing some plasma components, such as antibodies, immune complexes, endogenous and exogenous toxins, and replacing some proteins and coagulation factors [8]. In addition, severe HELLP syndrome with multiple organ failure has been suggested as a complication of thrombotic thrombocytopenic purpura and hemolytic uremic syndrome (TTP-HUS) or TTP-HUS itself [12, 13]. PE therapy has been established as a treatment for TTP and may effectively treat HELLP syndrome [14]. The decision to initiate PE therapy should be based on the severity of abnormalities, such as thrombocytopenia, and not on the diagnosis [13]. In our case, the patient also presented with symptoms of TTP, such as altered consciousness, which could be considered pregnancy-associated TMA, and PE therapy might have been effective.

## Conclusions

We report a case of postpartum HELLP syndrome that responded well to early PE therapy. PE therapy can be considered a crucial treatment for severe HELLP syndrome. The early initiation of PE therapy may be effective for early recovery, and early decisions should be made.

## Supplementary Information

**Additional file 1: Figure S1.** At 36 weeks and 6 days of gestation, due to rapidly progressing pulmonary edema and renal impairment with a serum creatinine of 0.90 mg/dL.

**Additional file 2: Supplemental Table 1.** Changes in coagulation values.

**Additional file 3: Supplemental Table 2.** Mississippi classification for classifying patients with HELLP syndrome.

## Data Availability

Not applicable.

## Abbreviations

AST: Aspartate aminotransferase
ALT: Alanine aminotransferase
CT: Computed tomography
DIC: Disseminated intravascular coagulation
HDP: Hypertensive disorders of pregnancy
HELLP syndrome: Hemolysis, elevated liver enzymes, and low platelets syndrome
HUS: Hemolytic uremic syndrome
ICU: Intensive care unit
LDH: Lactate dehydrogenase
PE: Plasma exchange
TMA: Thrombotic microangiopathy
TTP: Thrombotic thrombocytopenic purpura
