# Supplementary figures and images for: Therapeutic plasma exchange in postpartum HELLP syndrome: a case report
Source: JA Clin Rep. 2023 Feb 20;9:9. doi: 10.1186/s40981-023-00602-2 (PMC9939561; doi:10.1186/s40981-023-00602-2)

# Supplemental Figure 1

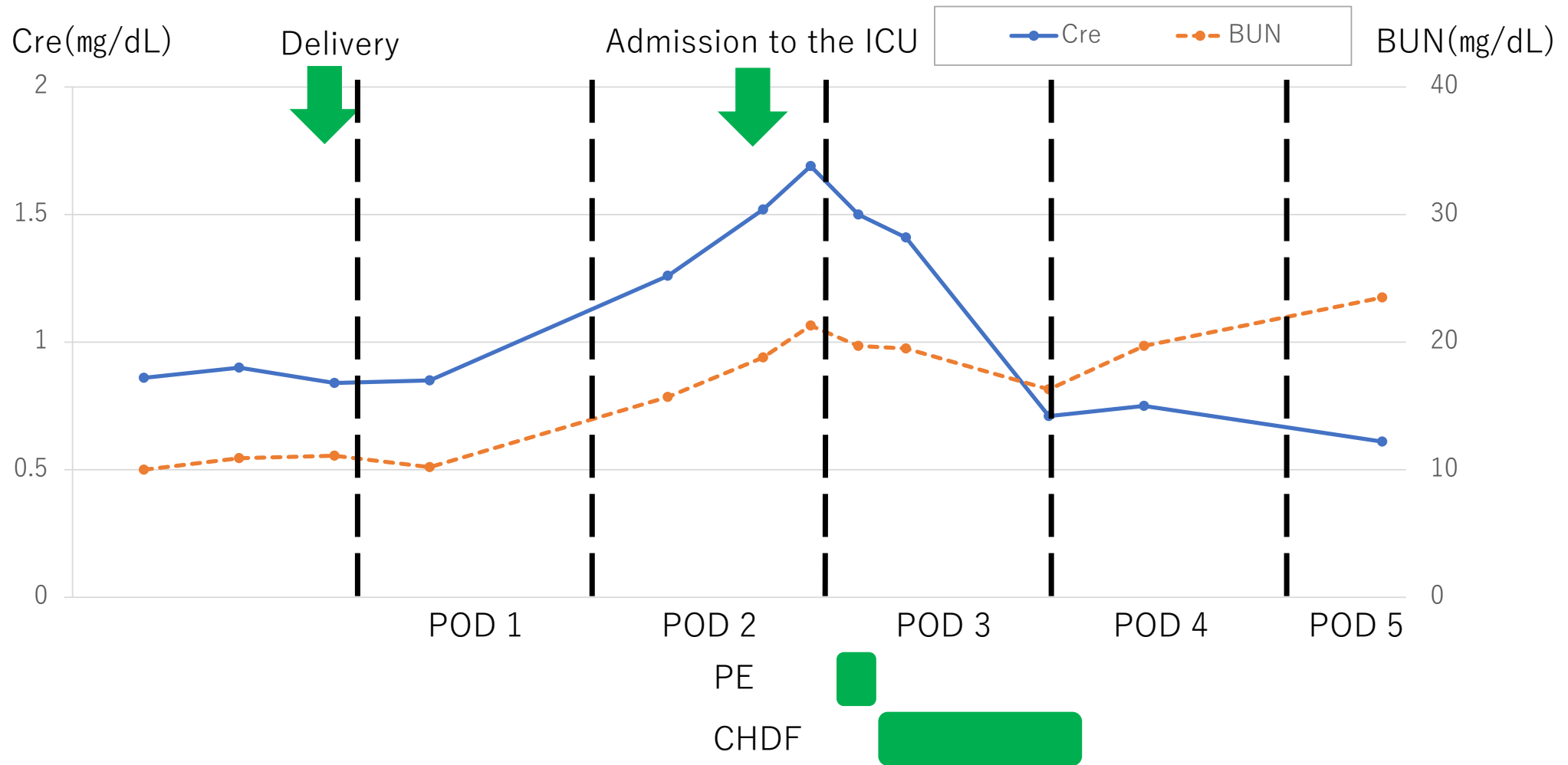

Supplement: Supplementary file 1 — Additional file 1: Figure S1. At 36 weeks and 6 days of gestation, due to rapidly progressing pulmonary edema and renal impairment with a serum creatinine of 0.90 mg/dL. [file 40981_2023_602_MOESM1_ESM.pdf]
