# Supplementary material for: Therapeutic plasma exchange in postpartum HELLP syndrome: a case report
Source: JA Clin Rep. 2023 Feb 20;9:9. doi: 10.1186/s40981-023-00602-2 (PMC9939561; doi:10.1186/s40981-023-00602-2)
Supplement: Supplementary file 2 — Additional file 2: Supplemental Table 1. Changes in coagulation values. [file 40981_2023_602_MOESM2_ESM.docx]

Supplemental Table 1

Changes in coagulation values.

| Variable | Preoperative | POD1 | POD2 | POD3 | | POD4 | POD5 |
| --- | --- | --- | --- | --- | --- | --- | --- |
|  |  |  | Pre ICU admission | Pre-PE | Post-PE |  |  |
| PT-INR | NA | 1.05 | 2.00 | 1.78 | 1.14 | 1.51 | 1.51 |
| APTT(sec) | NA | 40.0 | 34.0 | 38.9 | 38.1 | 34.1 | 27.8 |
| Fbg(mg/dL) | 397 | 211 | 97 | 154 | 239 | 176 | 142 |
| AT-III(%) | 62 | 42 | 34 | 96 | 78 | 72 | 85 |
| D-dimer  (µg/mL) | NA | NA | 28.2 | NA | 7.7 | 27.8 | 50.7 |

POD, post operative day. PE, plasma exchange. PT-INR, prothrombin time-international normalized ratio. APTT, activated partial thromboplastin time. Fbg, fibrinogen. AT-III, antithrombin III.
