# Supplementary material for: Therapeutic plasma exchange in postpartum HELLP syndrome: a case report
Source: JA Clin Rep. 2023 Feb 20;9:9. doi: 10.1186/s40981-023-00602-2 (PMC9939561; doi:10.1186/s40981-023-00602-2)
Supplement: Supplementary file 3 — Additional file 3: Supplemental Table 2. Mississippi classification for classifying patients with HELLP syndrome. [file 40981_2023_602_MOESM3_ESM.docx]

**Supplemental Table 2**

Mississippi classification for classifying patients with HELLP syndrome.

| Class | Platelets (/uL) | AST or ALT (IU/L) | LDH (IU/L) |
| --- | --- | --- | --- |
| I (severe) | ≤ 50,000 | ≥ 70 | ≥ 600 IU/L |
| II (moderate) | > 50000, ≤ 100000 | ≥ 70 | ≥ 600 IU/L |
| III (mild) | > 100000, ≤ 150000 | ≥ 40 | ≥ 600 IU/L |

ALT, alanine aminotransferase. AST, aspartate aminotransferase. HELLP, hemolysis, elevated liver enzymes, and low platelets. LDH, lactate dehydrogenase.
